# Correction: TreeSeq, a Fast and Intuitive Tool for Analysis of Whole Genome and Metagenomic Sequence Data

**DOI:** 10.1371/journal.pone.0131379

**Published:** 2015-06-22

**Authors:** Bastiaan B. Wintermans, Bernd W. Brandt, Christina M. J. E. Vandenbroucke-Grauls, Andries E. Budding

The authors’ names are listed incorrectly. The correct names are: Bastiaan B. Wintermans, Bernd W. Brandt, Christina M. J. E. Vandenbroucke-Grauls, and Andries E. Budding. The correct citation is: Wintermans BB, Brandt BW, Vandenbroucke-Grauls CMJE, Budding AE (2015) TreeSeq, a Fast and Intuitive Tool for Analysis of Whole Genome and Metagenomic Sequence Data. PLoS ONE 10(5): e0123851. doi:10.1371/journal.pone.0123851
