# Correction: TreeSeq, a Fast and Intuitive Tool for Analysis of Whole Genome and Metagenomic Sequence Data

**DOI:** 10.1371/journal.pone.0133816

**Published:** 2015-07-20

**Authors:** The PLOS ONE Staff

## Notice of Republication

This article was republished on July 2, 2015, to replace supporting information file S1 Software, which would not open. In addition, the republication addresses the errors in the author names noted in the correction published on June 22, 2015. The publisher apologizes for the errors. Please download this article again to view the correct version. The originally published, uncorrected article and the republished, corrected article are provided here for reference.

## Supporting Information

S1 File Originally published, uncorrected article. (PDF)

S2 File Republished, corrected article. (PDF)
