# Supplementary material for: TreeSeq, a Fast and Intuitive Tool for Analysis of Whole Genome and Metagenomic Sequence Data
Source: PLoS One. 2015 May 1;10(5):e0123851. doi: 10.1371/journal.pone.0123851 (PMC4416914; doi:10.1371/journal.pone.0123851)
Supplement: S1 Fig — Supplementary to Fig 1 this is an interactive Krona chart [8]. It details all the found results with BLAST for each accession-number in the ARDB-database within the metagenomic stool dataset (SRS022524.1). (HTML) [file pone.0123851.s002.html]

Javascript must be enabled to view this page.

magnitude
 64205
 12
 2
 1
 1
 10
 1
 1
 1
 1
 1
 1
 1
 1
 1
 1
 10
 2
 1
 1
 8
 4
 4
 3332
 2
 1
 1
 2623
 151
 151
 151
 151
 151
 124
 151
 151
 109
 125
 151
 151
 151
 151
 151
 151
 151
 151
 59
 17
 6
 6
 6
 18
 6
 161
 39
 31
 31
 60
 487
 62
 2
 20
 8
 17
 20
 62
 20
 20
 18
 20
 20
 16
 4
 2
 20
 20
 54
 20
 62
 12
 2
 1
 1
 10
 1
 1
 1
 1
 1
 1
 1
 1
 1
 1
 24
 2
 1
 1
 22
 1
 1
 1
 1
 1
 1
 1
 1
 1
 1
 1
 1
 1
 1
 1
 1
 1
 1
 1
 1
 1
 1
 1605
 1605
 185
 162
 185
 171
 162
 185
 185
 185
 185
 254
 254
 11
 12
 12
 11
 12
 11
 8
 12
 12
 12
 12
 12
 11
 11
 12
 12
 12
 12
 12
 12
 11
 12
 254
 254
 11
 12
 12
 11
 12
 11
 8
 12
 12
 12
 12
 12
 11
 11
 12
 12
 12
 12
 12
 12
 11
 12
 254
 254
 11
 12
 12
 11
 12
 11
 8
 12
 12
 12
 12
 12
 11
 11
 12
 12
 12
 12
 12
 12
 11
 12
 254
 254
 11
 12
 12
 11
 12
 11
 8
 12
 12
 12
 12
 12
 11
 11
 12
 12
 12
 12
 12
 12
 11
 12
 254
 254
 11
 12
 12
 11
 12
 11
 8
 12
 12
 12
 12
 12
 11
 11
 12
 12
 12
 12
 12
 12
 11
 12
 254
 254
 11
 12
 12
 11
 12
 11
 8
 12
 12
 12
 12
 12
 11
 11
 12
 12
 12
 12
 12
 12
 11
 12
 254
 254
 11
 12
 12
 11
 12
 11
 8
 12
 12
 12
 12
 12
 11
 11
 12
 12
 12
 12
 12
 12
 11
 12
 254
 254
 11
 12
 12
 11
 12
 11
 8
 12
 12
 12
 12
 12
 11
 11
 12
 12
 12
 12
 12
 12
 11
 12
 254
 254
 11
 12
 12
 11
 12
 11
 8
 12
 12
 12
 12
 12
 11
 11
 12
 12
 12
 12
 12
 12
 11
 12
 22
 22
 1
 1
 1
 1
 1
 1
 1
 1
 1
 1
 1
 1
 1
 1
 1
 1
 1
 1
 1
 1
 1
 1
 87
 87
 1
 14
 72
 5749
 668
 316
 352
 131
 20
 50
 27
 18
 16
 4950
 402
 339
 321
 389
 361
 422
 435
 395
 389
 361
 352
 389
 395
 102
 86
 2
 2
 2
 2
 2
 2
 2
 6
 2
 2
 2
 2
 6
 2
 2
 2
 2
 2
 2
 2
 2
 2
 2
 2
 2
 5
 2
 2
 2
 2
 7
 2
 2
 2
 2
 16
 3
 1
 1
 1
 7
 1
 1
 1
 56
 26
 1
 1
 1
 1
 1
 1
 1
 1
 1
 1
 1
 1
 1
 1
 1
 1
 1
 1
 1
 1
 1
 1
 1
 1
 1
 1
 30
 1
 1
 1
 1
 1
 1
 1
 1
 1
 1
 1
 1
 1
 1
 1
 1
 1
 1
 1
 1
 1
 1
 1
 1
 1
 1
 1
 1
 1
 1
 2843
 2623
 151
 151
 151
 151
 151
 124
 151
 151
 109
 125
 151
 151
 151
 151
 151
 151
 151
 151
 59
 17
 6
 6
 6
 18
 6
 161
 39
 31
 31
 60
 2843
 2623
 151
 151
 151
 151
 151
 124
 151
 151
 109
 125
 151
 151
 151
 151
 151
 151
 151
 151
 59
 17
 6
 6
 6
 18
 6
 161
 39
 31
 31
 60
 15
 15
 1
 1
 1
 1
 1
 1
 1
 1
 1
 1
 1
 1
 1
 1
 1
 23
 15
 1
 1
 1
 1
 1
 1
 1
 1
 1
 1
 1
 1
 1
 1
 1
 8
 4
 4
 22
 22
 1
 1
 1
 1
 1
 1
 1
 1
 1
 1
 1
 1
 1
 1
 1
 1
 1
 1
 1
 1
 1
 1
 22
 22
 1
 1
 1
 1
 1
 1
 1
 1
 1
 1
 1
 1
 1
 1
 1
 1
 1
 1
 1
 1
 1
 1
 10
 10
 1
 1
 1
 1
 1
 1
 1
 1
 1
 1
 52
 10
 1
 1
 1
 1
 1
 1
 1
 1
 1
 1
 8
 4
 4
 16
 3
 1
 1
 1
 7
 1
 1
 1
 16
 1
 5
 5
 5
 2
 1
 1
 45002
 10
 1
 1
 1
 1
 1
 1
 1
 1
 1
 1
 527
 106
 20
 31
 34
 34
 34
 34
 34
 20
 34
 20
 20
 106
 37
 10
 8
 12
 7
 1145
 12
 18
 13
 12
 17
 10
 17
 17
 18
 6
 13
 17
 20
 10
 10
 11
 12
 16
 19
 7
 17
 18
 16
 17
 17
 18
 10
 5
 18
 11
 8
 10
 10
 11
 10
 10
 10
 8
 12
 5
 17
 10
 13
 17
 14
 16
 12
 7
 18
 13
 11
 17
 13
 16
 11
 10
 17
 11
 1
 17
 17
 13
 17
 17
 10
 17
 12
 17
 11
 8
 6
 17
 19
 1
 17
 10
 10
 8
 18
 14
 12
 12
 18
 19
 18
 13
 17
 6819
 217
 217
 176
 206
 203
 270
 206
 180
 121
 187
 187
 159
 146
 180
 217
 154
 154
 146
 217
 182
 171
 203
 181
 180
 201
 200
 146
 217
 176
 150
 140
 217
 217
 148
 189
 187
 171
 26536
 1934
 1845
 294
 916
 301
 360
 413
 1845
 1934
 1934
 1845
 1845
 1845
 1845
 1845
 1845
 1845
 1845
 5
 5
 9923
 368
 150
 211
 393
 188
 241
 393
 393
 208
 107
 245
 125
 376
 218
 393
 8
 393
 379
 393
 120
 184
 159
 381
 211
 211
 393
 184
 188
 289
 188
 220
 3
 393
 163
 241
 211
 111
 182
 211
 310
 188
 8
 8
 4
 4
 68
 6
 2
 2
 2
 1
 1
 10
 5
 5
 30
 22
 8
 21
 7
 7
 7
