# Supplementary material for: TreeSeq, a Fast and Intuitive Tool for Analysis of Whole Genome and Metagenomic Sequence Data
Source: PLoS One. 2015 May 1;10(5):e0123851. doi: 10.1371/journal.pone.0123851 (PMC4416914; doi:10.1371/journal.pone.0123851)
Supplement: S2 Fig — Supplementary to Fig 1 this is an interactive Krona chart [8]. It details all the found results with TreeSeq for each accession-number in the ARDB-database within the metagenomic stool dataset (SRS022524.1). (HTML) [file pone.0123851.s003.html]

Javascript must be enabled to view this page.

magnitude
 32881
 748
 748
 85
 79
 85
 80
 79
 85
 85
 85
 85
 83
 83
 3
 4
 4
 3
 4
 3
 3
 4
 4
 4
 4
 4
 4
 3
 4
 4
 4
 4
 4
 4
 4
 4
 83
 83
 3
 4
 4
 3
 4
 3
 3
 4
 4
 4
 4
 4
 4
 3
 4
 4
 4
 4
 4
 4
 4
 4
 83
 83
 3
 4
 4
 3
 4
 3
 3
 4
 4
 4
 4
 4
 4
 3
 4
 4
 4
 4
 4
 4
 4
 4
 83
 83
 3
 4
 4
 3
 4
 3
 3
 4
 4
 4
 4
 4
 4
 3
 4
 4
 4
 4
 4
 4
 4
 4
 83
 83
 3
 4
 4
 3
 4
 3
 3
 4
 4
 4
 4
 4
 4
 3
 4
 4
 4
 4
 4
 4
 4
 4
 83
 83
 3
 4
 4
 3
 4
 3
 3
 4
 4
 4
 4
 4
 4
 3
 4
 4
 4
 4
 4
 4
 4
 4
 83
 83
 3
 4
 4
 3
 4
 3
 3
 4
 4
 4
 4
 4
 4
 3
 4
 4
 4
 4
 4
 4
 4
 4
 83
 83
 3
 4
 4
 3
 4
 3
 3
 4
 4
 4
 4
 4
 4
 3
 4
 4
 4
 4
 4
 4
 4
 4
 83
 83
 3
 4
 4
 3
 4
 3
 3
 4
 4
 4
 4
 4
 4
 3
 4
 4
 4
 4
 4
 4
 4
 4
 22
 22
 1
 1
 1
 1
 1
 1
 1
 1
 1
 1
 1
 1
 1
 1
 1
 1
 1
 1
 1
 1
 1
 1
 12
 12
 3
 9
 2678
 259
 124
 135
 41
 7
 11
 10
 7
 6
 2378
 191
 166
 165
 191
 175
 192
 194
 182
 191
 180
 178
 191
 182
 1515
 1401
 80
 80
 80
 80
 80
 68
 80
 80
 63
 70
 80
 80
 80
 80
 80
 80
 80
 80
 40
 9
 6
 6
 4
 11
 4
 74
 19
 16
 14
 25
 1515
 1401
 80
 80
 80
 80
 80
 68
 80
 80
 63
 70
 80
 80
 80
 80
 80
 80
 80
 80
 40
 9
 6
 6
 4
 11
 4
 74
 19
 16
 14
 25
 1753
 1401
 80
 80
 80
 80
 80
 68
 80
 80
 63
 70
 80
 80
 80
 80
 80
 80
 80
 80
 40
 9
 6
 6
 4
 11
 4
 74
 19
 16
 14
 25
 238
 3
 3
 3
 22
 2
 3
 3
 7
 6
 3
 3
 3
 9
 7
 22
 3
 3
 7
 9
 3
 9
 7
 7
 7
 3
 3
 3
 1
 3
 7
 9
 3
 15
 3
 3
 7
 22
 2
 49
 49
 1
 1
 1
 1
 1
 1
 1
 1
 1
 1
 1
 1
 1
 1
 1
 1
 1
 1
 1
 1
 1
 1
 1
 1
 1
 1
 1
 1
 1
 1
 1
 1
 1
 1
 1
 1
 1
 1
 1
 1
 1
 1
 1
 1
 1
 1
 1
 1
 1
 23825
 257
 53
 12
 13
 15
 15
 15
 15
 15
 12
 15
 12
 12
 53
 14
 4
 3
 4
 3
 726
 7
 11
 8
 7
 10
 6
 11
 11
 11
 4
 8
 11
 11
 6
 6
 6
 8
 10
 11
 4
 11
 11
 11
 11
 11
 11
 6
 3
 11
 6
 5
 6
 6
 6
 6
 6
 6
 8
 8
 3
 11
 6
 8
 11
 11
 10
 9
 4
 11
 9
 6
 11
 8
 10
 6
 6
 11
 7
 1
 11
 11
 9
 11
 11
 10
 11
 6
 10
 8
 5
 5
 10
 11
 1
 11
 6
 10
 5
 11
 11
 8
 7
 11
 11
 11
 8
 11
 3611
 109
 109
 99
 104
 106
 123
 104
 95
 78
 98
 100
 97
 81
 95
 109
 84
 84
 81
 109
 95
 98
 105
 103
 95
 102
 101
 81
 109
 99
 88
 76
 109
 109
 82
 97
 100
 97
 13543
 938
 907
 279
 533
 285
 253
 309
 907
 938
 938
 907
 907
 907
 907
 907
 907
 907
 907
 2
 2
 5672
 190
 99
 129
 198
 125
 149
 198
 198
 126
 83
 141
 93
 192
 134
 198
 2
 198
 193
 198
 88
 115
 103
 191
 129
 129
 198
 115
 125
 153
 125
 136
 1
 198
 105
 149
 129
 82
 124
 129
 179
 125
 17
 6
 2
 2
 2
 2
 1
 1
 6
 5
 1
 3
 1
 1
 1
