# Supplementary figures and images for: TreeSeq, a Fast and Intuitive Tool for Analysis of Whole Genome and Metagenomic Sequence Data
Source: PLoS One. 2015 May 1;10(5):e0123851. doi: 10.1371/journal.pone.0123851 (PMC4416914; doi:10.1371/journal.pone.0123851)

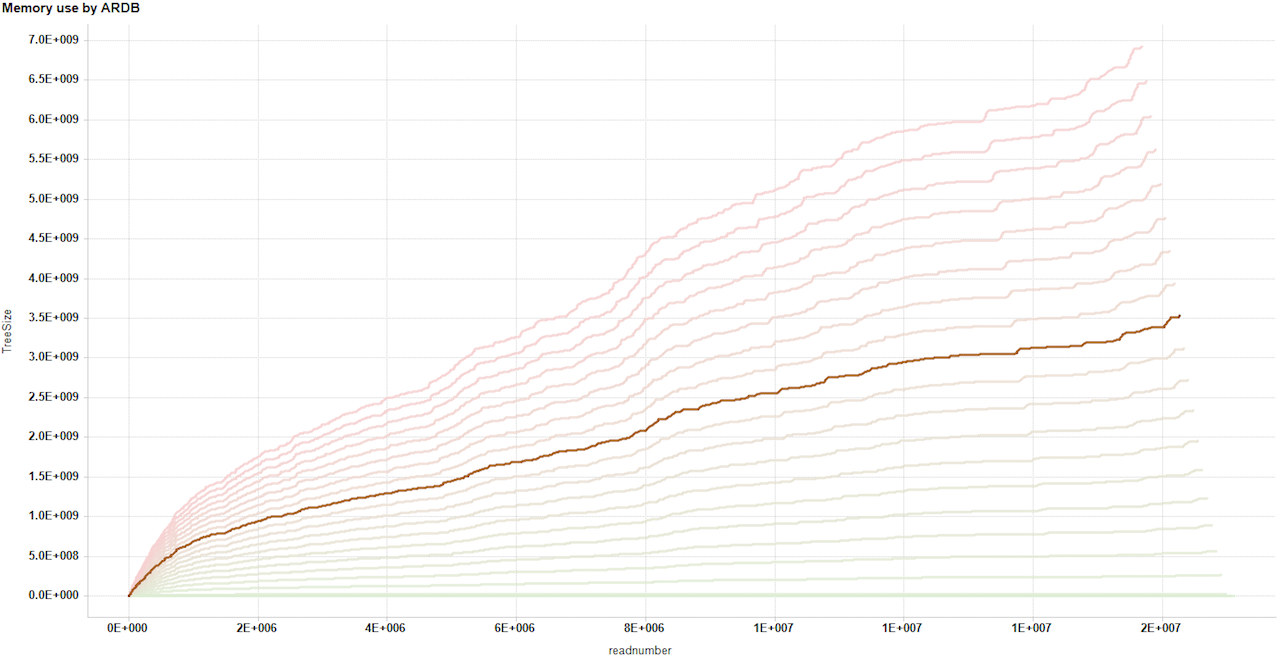

Supplement: S3 Fig — This graph represents the memory use while filling up the tree. On the x-axis is the number of entries derived from the gene sequences in the ARDB as described in the method section. On the y-axis is the memory footprint in bytes. The individual lines represent different read lengths, ranging from 1 (green) to 100 (red) nucleotides. The marked line in the middle represents read length of 60 nucleotides, used in this study. (TIFF) [file pone.0123851.s004.tiff]

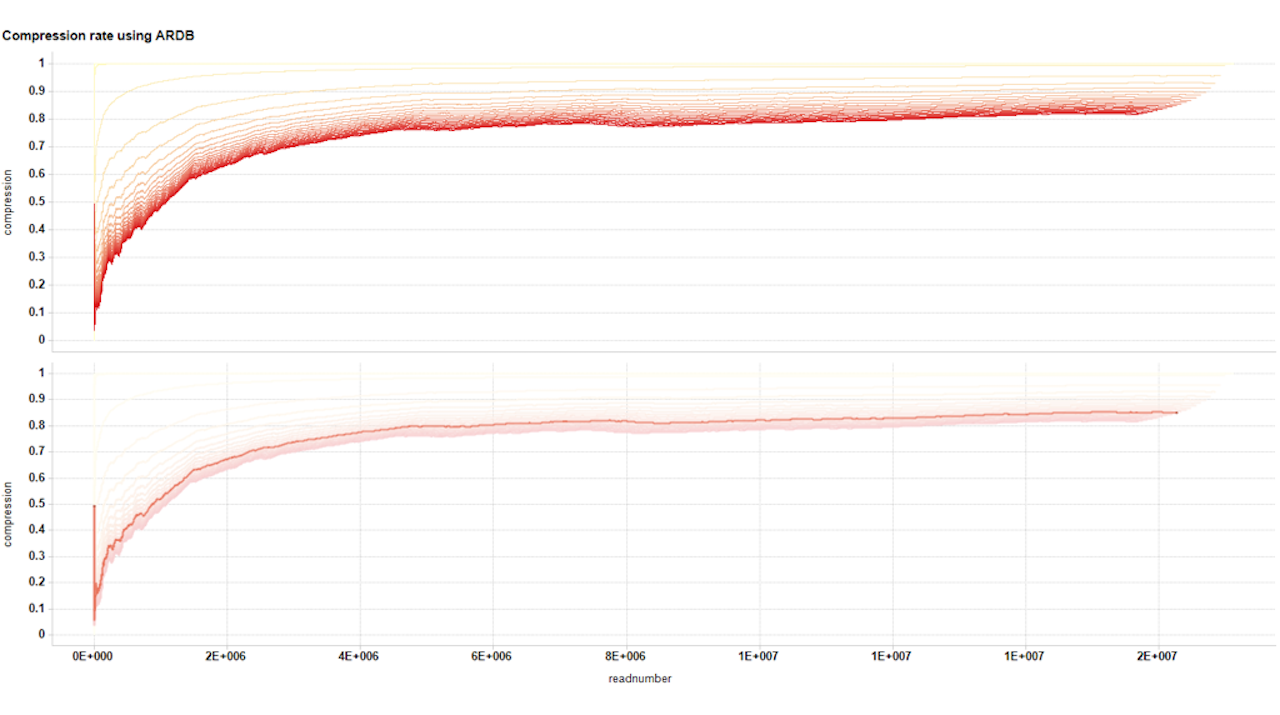

Supplement: S4 Fig — These graphs represent the data compression that occurs while filling up the tree, which occurs because of (partial) overlap in the nodes of the tree as described in the method section. On the x-axis is the number of entries derived from the gene sequences in the ARDB as described in the method section. On the y-axis is the compression rate. The individual lines represent different read lengths, ranging from 1 (yellow) to 100 (red) nucleotides. Compression rate = 1 - (((number of nodes in tree)/(read length)) * (sum of reads)) The lower graph is a duplicate of the upper graph with a marked line, which represents the read length of 60 nucleotides that we used for this study. (TIFF) [file pone.0123851.s005.tiff]
