# Supplementary material for: Correction: TreeSeq, a Fast and Intuitive Tool for Analysis of Whole Genome and Metagenomic Sequence Data
Source: PLoS One. 2015 Jul 20;10(7):e0133816. doi: 10.1371/journal.pone.0133816 (PMC4508097; doi:10.1371/journal.pone.0133816)
Supplement: S1 File — (PDF) [file pone.0133816.s001.pdf]

RESEARCH ARTICLE

TreeSeq, a Fast and Intuitive Tool for Analysis of Whole Genome and Metagenomic Sequence Data

Bastiaan Wintermans[1]*, Bernd Brandt[2], Christina Vandenbroucke-Grauls[1], Andries Budding[1]

1 Department of Medical Microbiology and Infection Control, VU University Medical Center, Amsterdam, The Netherlands, **2** Department of Preventive Dentistry, Academic Centre for Dentistry Amsterdam (ACTA), University of Amsterdam and VU University Amsterdam, Amsterdam, The Netherlands

* bb.wintermans@vumc.nl

Abstract

Next-generation sequencing is not yet commonly used in clinical laboratories because of a lack of simple and intuitive tools. We developed a software tool (TreeSeq) with a quaternary tree search structure for the analysis of sequence data. This permits rapid searches for sequences of interest in large datasets. We used TreeSeq to screen a gut microbiota metagenomic dataset and a whole genome sequencing (WGS) dataset of a strain of *Klebsiella pneumoniae* for antibiotic resistance genes and compared the results with BLAST and phenotypic resistance determination. TreeSeq was more than thirty times faster than BLAST and accurately detected resistance gene sequences in complex metagenomic data and resistance genes corresponding with the phenotypic resistance pattern of the Klebsiella strain. Resistance genes found by TreeSeq were visualized as a gene coverage heat map, aiding in the interpretation of results. TreeSeq brings analysis of metagenomic and WGS data within reach of clinical diagnostics.

OPEN ACCESS

Citation: Wintermans B, Brandt B, Vandenbroucke-Grauls C, Budding A (2015) TreeSeq, a Fast and Intuitive Tool for Analysis of Whole Genome and Metagenomic Sequence Data. PLoS ONE 10(5): e0123851. doi:10.1371/journal.pone.0123851

Academic Editor: Ahmed Moustafa, American University in Cairo, EGYPT

Received: September 29, 2014

Accepted: January 8, 2015

Published: May 1, 2015

Copyright: © 2015 Wintermans et al. This is an open access article distributed under the terms of the Creative Commons Attribution License, which permits unrestricted use, distribution, and reproduction in any medium, provided the original author and source are credited.

Data Availability Statement: All relevant data are within the paper and its Supporting Information files.

Funding: The authors have no support or funding to report.

Competing Interests: The authors have declared that no competing interests exist.

Introduction

Introduction of new genotypic methods in clinical diagnostics often leads to new diagnostic strategies. One of the most promising techniques at this moment, which is still not widely available in clinical laboratories, is next-generation sequencing (NGS). It has dramatically improved sequencing throughput and has made the field of shotgun metagenomics possible, where the genetic content of whole bacterial communities can be analysed without prior culture [1,2].

Unlike whole-genome shotgun sequencing (WGSS) of individual organisms, in which the end product is typically an assembled genome, metagenomics handles multiple genomes. This is why metagenomic data files are often large and highly complex; therefore analysis frequently requires high-end computers, which are not part of the routine equipment of clinical laboratories. To introduce NGS to these laboratories, we therefore need a rapid and user-friendly tool

that compares the reads to sequences of interest on a normal desktop computer and supports straightforward interpretation of the results.

We developed a software tool called TreeSeq that greatly simplifies the search task and generates accurate results from NGS datasets. Data visualization offers users an intuitive way to interpret the results and may therefore be easily used for NGS analysis in clinical laboratories.

A possible application of this tool is to search for antibiotic resistance genes in NGS shotgun metagenomics data. Accurate assessment of the often complex resistance patterns leads to better understanding of antimicrobial resistance and the epidemiology of the spread of antibiotic resistance. It is even conceivable that culture-independent antibiotic resistance tests may replace conventional methods in clinical microbiology laboratories in the future [1,2].

In this study, we use metagenomics and WGSS data to search for antimicrobial resistance genes, post-process the search results and visualize the reads mapping to a database of known resistance genes. First, we analysed stool sample data from the Human Microbiome Project (HMP) as proof of principle for searching genes and to demonstrate the analysis of metagenomic data [3,4]. The results of our TreeSeq program were compared to the widely used BLAST algorithm [5], which we took as golden standard for identification of gene sequences in metagenomics to check the performance of our tool.

In a second proof-of-principle setup, we used TreeSeq to detect antimicrobial resistance genes in a complete whole genome shotgun sequence set of a multidrug-resistant strain of *Klebsiella pneumonia* and compare this to the current standard in susceptibility testing which is culture based. The genetic resistance pattern thus obtained was compared to the phenotypical antibiogram. In bacterial strains little is known about the expression of these genotypic patterns. This makes a comparison to culture essential prior to predicting the applicability of TreeSeq to the daily practice in clinical laboratories.

Results

TreeSeq versus BLAST in HMP stool data

We performed a head-to-head comparison of BLAST and TreeSeq by using the two algorithms on the same metagenomic dataset (SRS022524.1.fastq) to searching for the resistance genes of the Antibiotic Resistance Genes Database (ARDB) [5,6,7]. For details on BLAST, TreeSeq and ARDB we refer to the methods section.

BLAST found 492 genes, comprising 64205 (1–1934, Avg. 76) search hits from the ARDB that corresponded to 34 resistance gene classes in the HMP metagenomic stool data. TreeSeq found 401 genes, comprising 32881 (1–938, Avg 52) hits corresponding to 23 resistance gene classes. The 11 gene classes missed with TreeSeq all had a hit occurrence of less then seven hits. TreeSeq did not detect genes that were not found by BLAST. Fig 1 gives an overview of these results. S1 Fig and S2 Fig show the results for BLAST and TreeSeq in interactive Krona charts [8].

TreeSeq versus Phenotypical tests

The phenotypic analysis of *K. pneumoniae* (BAA-2146), as provided by American Type Culture Collection (ATCC), shows that this strain is resistant to 35 antimicrobials. The WGSS of this reference phenotype was used for the comparison to TreeSeq [9,10]. S1 Table shows the phenotype according to ATCC.

In the WGSS dataset, available from NCBI (NZ_AOCV01000027.1), TreeSeq found genes conferring resistance to 31 of these 35 antimicrobials. TreeSeq missed resistance to: amoxicillin/clavulanic acid, ticarcillin/clavulanic acid, ampicillin/sulbactam and nitrofurantoin. The reason for this was that the genes corresponding to resistance for beta-lactamase inhibitors and

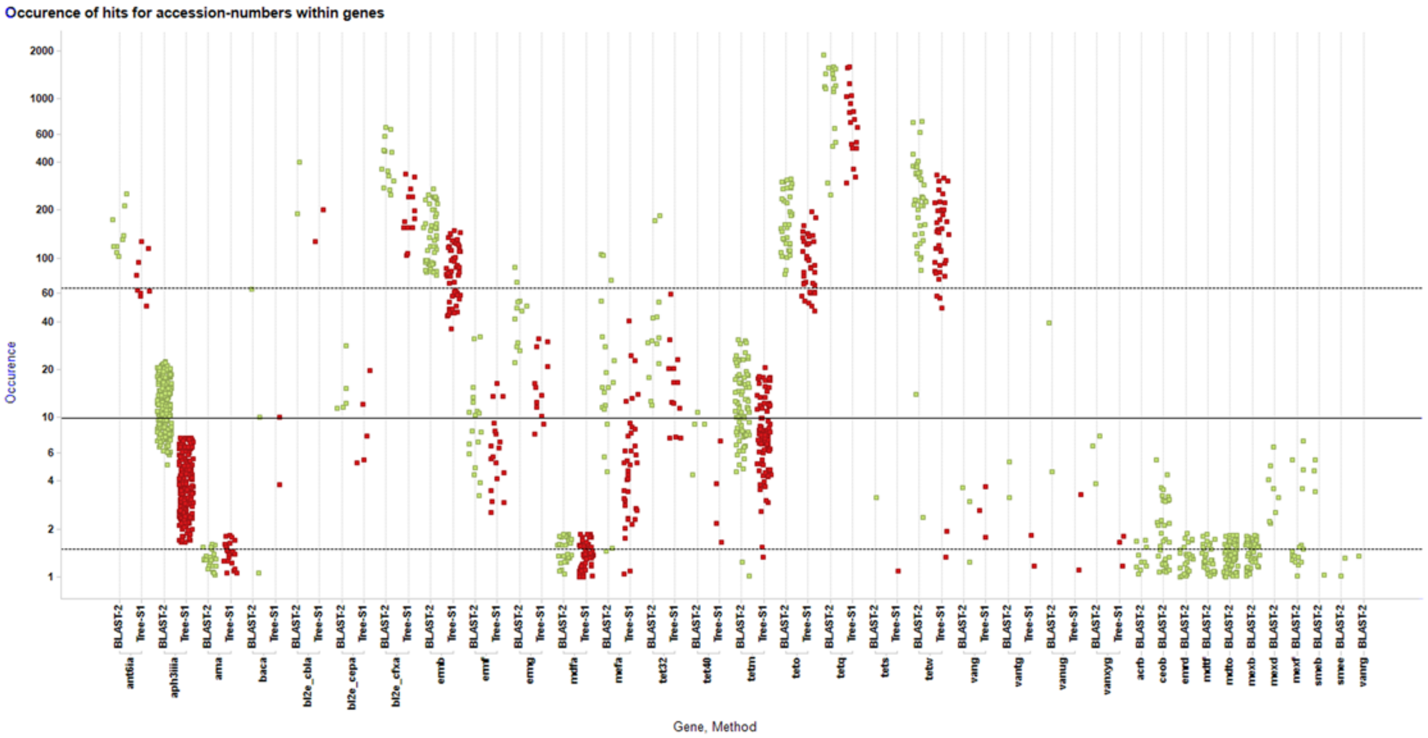

Fig 1. TreeSeq versus BLAST. This scatterplot shows the results for the two methods, BLAST (green) versus TreeSeq (red), by searching metagenomic stool dataset SRS022524.1. On the x-axis all the found gene classes and for each gene class the results per method are shown. On the y-axis the hit occurrences, in log scale, for each gene is shown as a dot. On the far right the results are shown that were only found by BLAST, here the highest occurrence was 7 hits.

doi:10.1371/journal.pone.0123851.g001

nitrofurantoin were not present in the ARDB. TreeSeq found 16 genes in the whole genome shotgun sequence of *K. pneumoniae* that could result in resistance of which the corresponding phenotypic resistance data were not provided by ATCC. Fig 2 and S1 Table gives an overview of the resistance genes reported and found by TreeSeq.

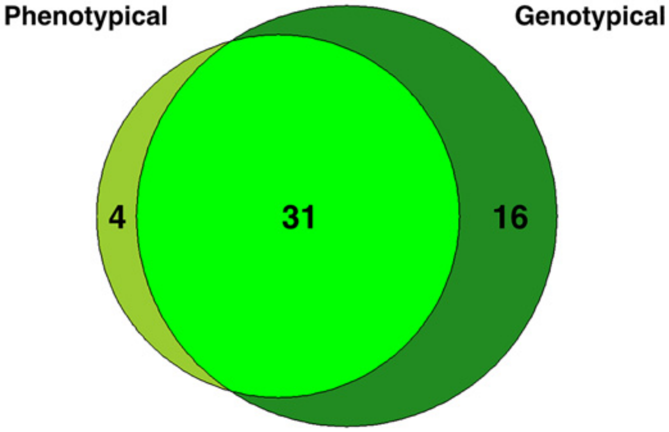

Fig 2. TreeSeq versus phenotype. American Type Culture Collection (ATCC) tested resistance in *Klebsiella pneumonia* (BAA-2146), which is a phenotypical referenced strain, by culturing. This diagram shows result overlap for phenotypical resistance testing versus genotypical testing with TreeSeq using the ARDB.

doi:10.1371/journal.pone.0123851.g002

Visualisation and interpretation of TreeSeq data

The TreeSeq results were visualized with a heat map (Spotfire, TIBCO, Palo Alto), which allows a straightforward visual assessment and interpretation of found/uncovered resistance genes. The heat map of the individual genes shows the occurrence of the 60-nucleotide long read fragments detected within that gene. High occurrence along a reference sequence implies that the whole gene was found multiple times. Low occurrence in a small segment implies that only a partial nucleotide sequence matching this reference gene was found. Fig 3 shows the resistome of *Klebsiella pneumonia* (BAA-2146) [10]. Fig 4 shows the metagenomics-based resistome of the stool sample (SRS022524.1) [7].

Discussion

In clinical laboratories, a user-friendly tool, able to run on a desktop computer, is needed that can find sequences of interest in NGS-data. We created TreeSeq, a tool that meets both these requirements. TreeSeq uncovers specific genes in NGS-data, using any reference database as input for the search task.

TreeSeq structures a database to searchable trees and then compares the NGS reads to the nodes in the tree in a stepwise fashion. This allows for fast searching, since the search task never takes more steps than the length of the tree. This greatly simplifies the search process and in addition does not require high-end computer performance. Visualisation of the

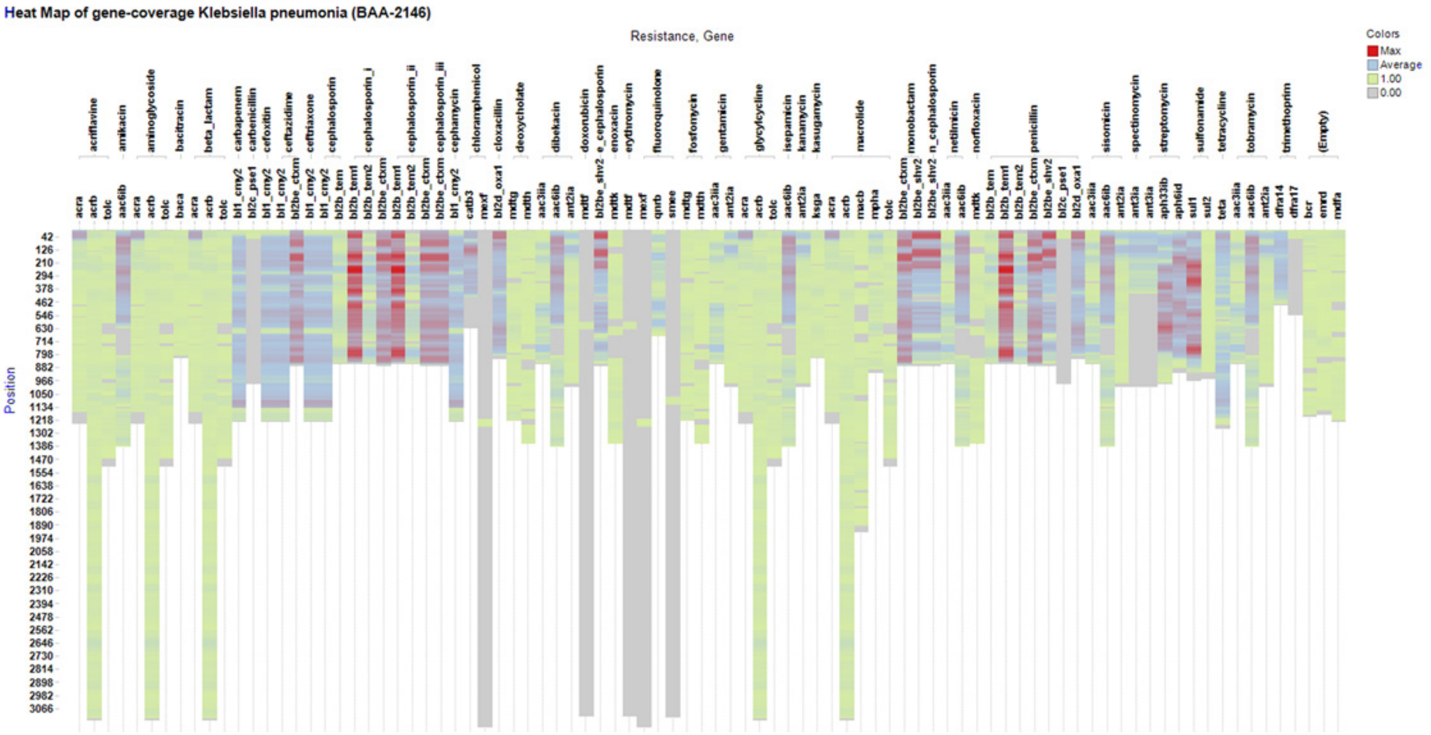

Fig 3. TreeSeq results of *Klebsiella pneumonia*. This is a heat map of the results generated by TreeSeq of the *Klebsiella pneumonia* (BAA-2146) strain. On the x-axis the found resistance gene classes and the antibiotic that it confers resistance to. It is possible to increase the search result resolution by adding the specific gene level, which is not displayed. The y-axis shows the nucleotide positions of the gene and therefore the length of the bar also represents the length of the gene. The colour represents the amount of occurrences (hits) of a gene on this specific position.

doi:10.1371/journal.pone.0123851.g003

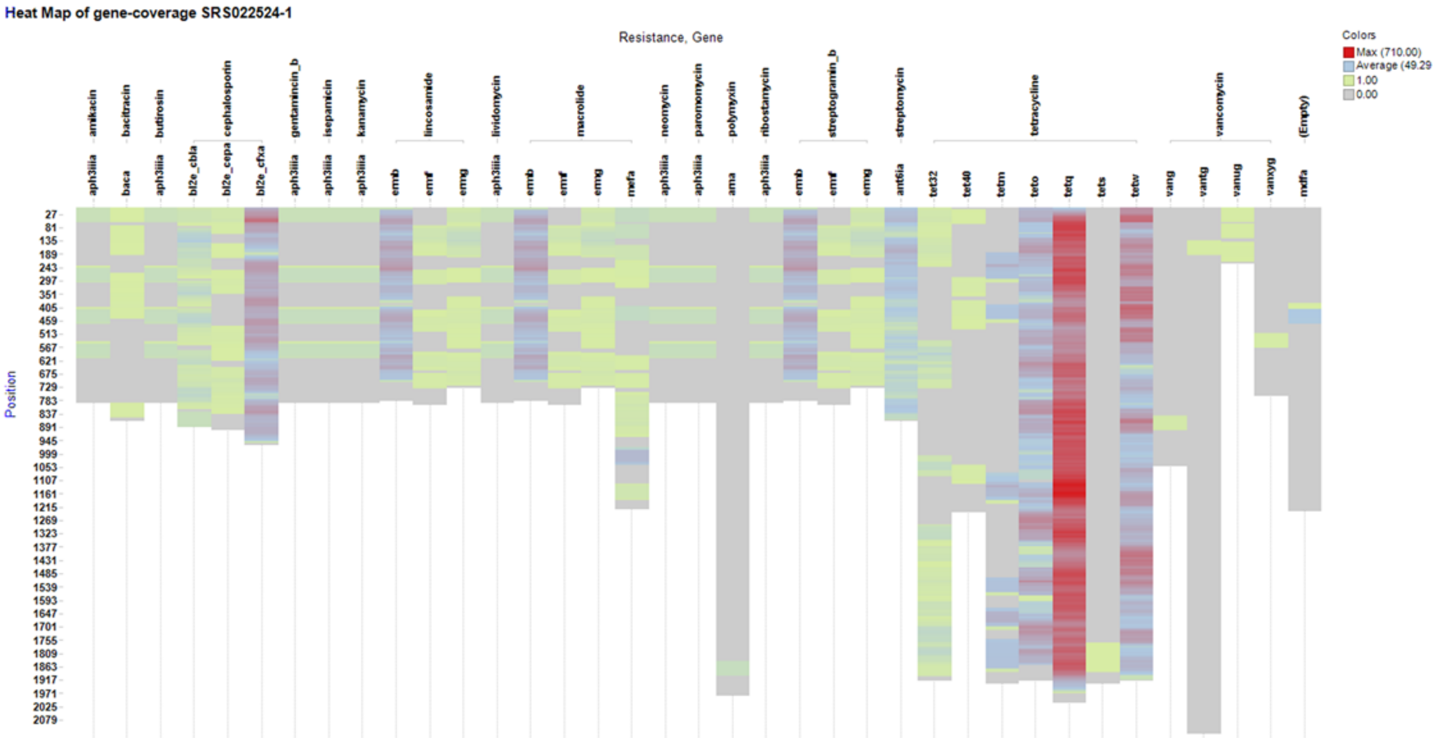

Fig 4. TreeSeq results of metagenomic stool dataset. This is a heat map of the results generated by TreeSeq of the metagenomic stool dataset (SRS022524.1). On the x-axis the found resistance gene classes and the antibiotic that it confers resistance to. It is possible to increase the search result resolution by adding the specific gene level, which is not displayed. The y-axis shows the nucleotide positions of the gene and therefore the length of the bar also represents the length of the gene. The colour represents the amount of occurrences (hits) of a gene on this specific position.

doi:10.1371/journal.pone.0123851.g004

results makes interpretation much easier and could lead to the accuracy needed for making clinical decisions.

What could be regarded as a drawback of TreeSeq is that only identical matches can be found. However, this only applies to individual reads. When read matches are combined, as is done for the visualization, missing individual reads is not so relevant. Due to the relatively high coverage in the data sets, multiple reads of the same gene are expected to be present. When only one or a few reads are found in a specific gene, the specificity of these findings should be questioned, as these can relate to sequencing errors or aspecific matches, and may lead to erroneous interpretations. For clinical interpretation therefore, single copy findings should be ignored, thus reducing the sensitivity constraints. A second reason for emphasizing the importance of exact matches of reads, is that many phenotypic antibiotic resistance patterns may be conferred by single point mutations (e.g. β-lactamases) [11]. As with sequence errors, also low quality gene catalogues will result in loss of sensitivity. Many publicly available gene catalogues nowadays contain good quality sequences and will detect relevant genes in TreeSeq as demonstrated.

In this study, we propose the use of TreeSeq to search for resistance genes in both WGSS and in shotgun metagenomics data. We compared TreeSeq to the BLAST algorithm to demonstrate the efficacy of our tool. Subsequently, we use TreeSeq for prediction of phenotypical resistance in which we find the only limiting factor to be the content of the resistance gene database that is used.

We tested TreeSeq to search metagenomic data that was generated by the HMP. With BLAST, we searched for over 7000 (3571 non-redundant) resistance genes and found reads corresponding to almost 500 resistance genes. TreeSeq found less, namely 82%, of the sequences corresponding to well-known resistance gene sequences. To put this in perspective, all these "missed" results were low frequency hits and never exceeded 7 hits per gene. It is conceivable that low frequency hits in BLAST occur more often due to sequence similarity of reads with other genes. In this case, the high sensitivity of BLAST leads to multiple hits in various genes. The occurrence of these hits can be partially filtered by demanding high E-values and high percent identity thresholds. Alternatively, the BLAST results could be post-processed, based on relevance and specificity criteria using enveloping software. However, even with these additions, the significantly slower performance of the BLAST algorithm and its lack of integrated post-processing makes the quaternary tree approach of TreeSeq more practical in a clinical routine setting.

Visualizing the results in a gene heat map allows users to interpret the results. The means to do this is integral to the tree structure, as gene characteristics, such as name and coordinates in the reference sequence, are linked to the end nodes in the tree. By storing occurrences in all reference genes of every read in the associated end node, it is directly apparent if a read is specific (occurrence in only one gene) or aspecific (occurrence in multiple genes). Visualizing the coverage of identified resistance genes along their sequence makes assessment of gene abundances fast, accurate and easier to interpret. For example, it is highly unlikely that in one gene of 1200 nucleotides, only one small sequence of 60 nucleotides occurs multiple times. It is more likely that this is due to sequence similarity of a read to another gene. In other words, genes that are abundant in a sample have a more equal distribution of their related reads along the reference sequence. Total sequence coverage along the full length of the gene makes it very plausible that the complete gene really is present in the NGS data.

TreeSeq also proved to be accurate in detecting antimicrobial resistance genes in WGSS data of individual strains. We used TreeSeq to scan a complete WGSS dataset of a multidrug-resistant strain of *K. pneumonia* for resistance genes and found the genes that corresponded to the phenotypic resistance pattern, except for those genes that were not present in the ARDB. The limitation in finding (specific) resistance traits lies in the quality of the genetic resistance database: the more complete the list of resistance genes, the better the results that will be obtained with TreeSeq. Since NGS protocols are becoming easier and prices are still dropping, the time is near that WGS can be introduced in clinical laboratories for use on a daily basis.

Metagenomics has greatly increased our knowledge of the composition of the human microbiota and has allowed the identification of microorganisms that were previously unrecognized [3,4]. Gaining experience in searching for clinical relevant genes and uncovering their abundance in metagenomics or WGSS data may also lead to new insights for clinicians. Besides the detection of specific resistance genes in WGSS data, interesting applications can be detection of pathogenicity islands, virulence factors and genes that promote their expression. In metagenomic analysis, applications can be envisaged for genotyping of microorganisms or their genes.

With the rapid expansion of publicly available gene catalogues, sequences of specific resistance genes of clinically important bacteria will increasingly become available. Identifying these species-specific resistance genes in metagenomic data is highly desirable for clinical applications. In order to pinpoint the subtle genomic variation that may be used to assign a resistance gene to a specific bacterial species, matches to database entries should be as identical as possible. We argue that our proposed approach with TreeSeq is highly suitable for a clinical setting, as it delivers the needed specificity at a very high speed. Through post-processing, aspecific reads can be redirected towards genes for which specific hits have also been found. This feature of TreeSeq provides more specific results, which is essential for prediction of phenotypical antibacterial resistance profiles.

Conclusion

TreeSeq structured gene data efficiently and made fast and accurate searches possible, without the need for large computing power. This made it possible to easily search for genes of interest; results were visualised for easy interpretation. TreeSeq differs from BLAST by higher speed and precision through post-processing, with a small trade-off in sensitivity. Precision is needed to detect single point mutations, which can be essential for clinical application. As proof-of-principle, we demonstrated the use of TreeSeq to search for resistance genes to construct a resistome heat map and correlated resistance genes to their phenotype. Therefore, this tool appears well suited to bring NGS within reach of clinical laboratories.

Methods

Resistance genes

The Antibiotic Resistance Genes Database (ARDB) unifies information about a large variety of antibiotic resistance traits. Each protein and resistance type is linked to external protein databases [6]. A conversion was made from the ARDB protein database, supplied with ardbAnno1.0 (resisGenes.pfasta from ardbAnno1.0; 7828 records; 3002 non-redundant records) to a nucleotide database. The GenBank records of these proteins were downloaded from NCBI Entrez Proteins after which their coding sequences (CDS) were taken from the corresponding nucleotide record. The GenBank records of the Swiss-Prot and PDB accessions did not contain the CDS; the final nucleotide version of ARDB contained 7279 (3571 non-redundant) genes. The converted database can be downloaded as a fasta file from the supporting information section, S1 Database.

BLAST

Shotgun metagenome data was analysed against the ARDB gene set using megablast version 2.2.27+ with an identity score > = 90% [5,6]. For each read, all top-scoring hits in the ARDB genes were used for comparison with TreeSeq results. In case there was more than one top hit, all hits with equal E-value were included.

BLAST runs were performed on a server with a 12 cores (Intel Xeon L5640, 2.26 GHz), 24 GB memory and running 64-bit Linux. Runtime (wall-time) was 41 minutes for SRS022524.1 (10372511 reads) [7] against non-redundant ARDB gene set (3571 genes) [6].

TreeSeq

We designed software that analyses NGS read data. The script is C++ based and runs on a laptop computer, with a 2.3 GHz Intel Quad Core CPU i7, 16 GB 1600 MHz DDR3, running Mac OS X. In this study, we used 100 nucleotide reads obtained from the HMP for the analysis.

Runtime (wall-time) was 80 seconds, using only one thread, for SRS022524.1 (10372511 reads) [7] against non-redundant ARDB gene set (3571 genes) [6]. Our tool structures input data, which are gene sequences in FASTA format, in nodes along a quaternary tree for the search task (tree data structure is illustrated in Fig 5). The genes are cut along a sliding window of one nucleotide over the gene sequence to fragment the (reference) gene to consecutive subsequences of a specified length. For the present analysis, we used fragments of 60 nucleotides. Not only is this the shortest length of the reads in the raw data file, but more importantly it ensures precise mapping. All consecutive subsequences and the corresponding information about its originating gene, namely the name and its nucleotide-coordinates in the gene, are added to the end node of the tree during this process. This reduces effort spent on data overlap in the subsequences and makes rapid searches possible, because identification of a match always has a

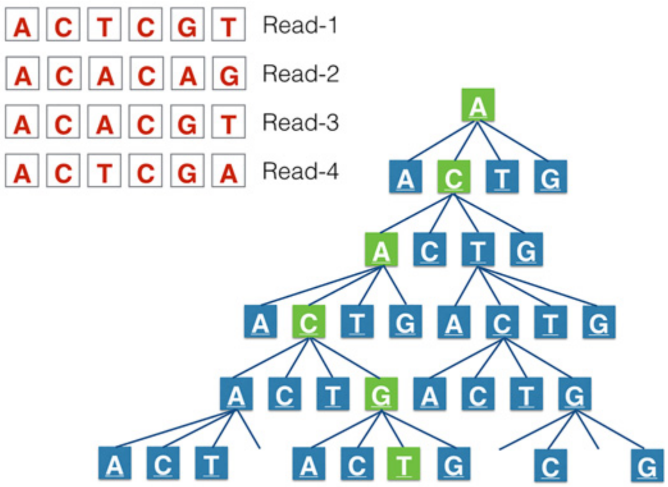

Fig 5. TreeSeq tree structure. This represents the quaternary tree in which all possible 60-nucleotide long read combinations of the resistance database were added. For the search task all (sub)reads in the NGS file are compared to the nodes in the tree. If the nodes in the tree can be run through to the last node in the tree (as in Read-3), this is a hit.

doi:10.1371/journal.pone.0123851.g005

maximum number of steps, which is equal to the length of the sequence as defined when generating the tree, regardless of the magnitude of the search database. The entire tree is loaded into memory. Extra data about the memory footprint can be found in the supporting information. The S3 Fig represents the memory footprint and the S4 Fig represents the data compression that occurs in the tree structure.

Once the data is structured, a comparison to the NGS reads can be made. Prior to this no assembly of reads is required. This means that only partial gene-sequences, for example, of 60 nucleotides can be found. Based on the software settings for optimal speed at cost of sensitivity, only the first 60 nucleotides of the raw read data can be used as input. When optimal sensitivity is needed every possible subsequence comprising the read can be used by using a sliding window of 60 nucleotides within the read. Each following nucleotide of these read subsequences is compared to each node in the binary tree. When the software is able to completely run through the tree to the last node, the software interprets this as a positive search result. During this process no mismatches are accepted, which will result in only exact hits. Searching for subsequences of a read is stopped when a hit occurs in it. This avoids distortion of hit distribution due to high abundant genes, which would cause high numbers of hit occurrences if searching within the read would be continued. In this study, we used the sensitive setting. The S5 Fig shows the distribution of the search results using the fast and accurate software configurations.

After searching, the hits in the reads can be assembled/mapped to output a complete gene using the stored nucleotide-coordinates from the end nodes of the reference sequences. An extra feature of the quaternary tree is the ability to correct for aspecific search results, which occur because of genetic resemblances between (resistance) genes within its sequence. After a read has a hit in a branch of the tree, the tree knows how many genes in the database correlate to the found read therefor it is possible to correct for aspecific hits. This is done, after a hit occurs, by counting the total amount of the stored genes with the correlating nucleotide-coordinates in the end nodes of the tree branch. If there is one gene in an end node the hit for this gene is specific in this database. If there are many possible gene hits in the end node the read is aspecific and it is possible to keep these results aside until specific reads for the genes are

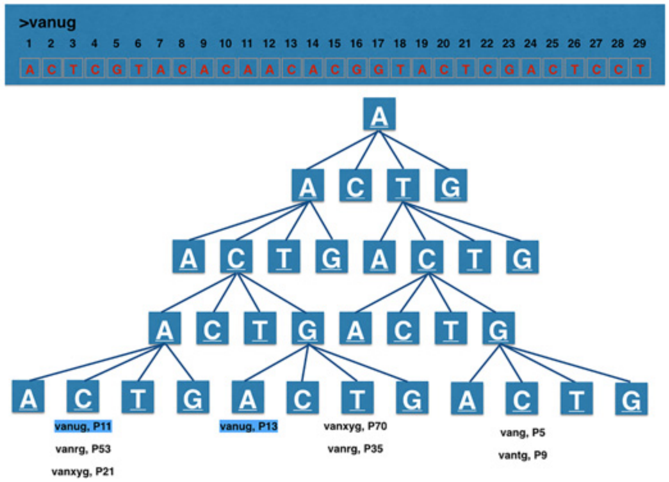

Fig 6. TreeSeq search result processing. This represents the quaternary tree in which all possible nucleotide read combinations of the resistance database were added. During this process the read's gene of origin and its nucleotide-coordinates were added to the end-nodes. If during a search task an end-node contains one gene, it is a specific result for one particular gene in the database. These obtained results can be copied to a result list. In case the end nodes contain multiple genes, this read is not specific for a gene. This aspecific result can be copied to a separate list, namely the doubtlist, for post processing. After comparing all the raw data reads to the tree, the software makes up the balance and looks for which genes in the doubtlist it already has specific hits in the result list and supplements them if so.

doi:10.1371/journal.pone.0123851.g006

found, if so the found 'aspecific' reads can be added to the found specific hits for that gene. Fig 6 demonstrates this feature.

A terminal based version of TreeSeq can be downloaded from the supporting information section, S1 Software.

Human Microbiome Project Data

The National Institutes of Health launched the HMP in 2007 with the mission to characterize the human microbiome and analyse its role in health and disease. Participants in the HMP were healthy adults, who did not receive antibiotics in the prior 6 months. Samples were obtained from various body sites, including the gastrointestinal tract. The DNA extracted from each sample was sequenced with Illumina NGS to generate raw data files, which are stored at the Illumina short read archive and made available freely for other researchers. Therefore, in this study, we did not work with any human tissues or samples. The data we used (SRS022524.1.fastq) for the comparison to BLAST was obtained from the HMP stool sample archive [3,4,7].

Phenotypical susceptibility

We used data publicly available at the American Type Culture Collection (ATCC). *Klebsiella pneumoniae* BAA-2146, tested by ATCC, shows phenotypic resistance to 35 antimicrobial agents and antimicrobial/inhibitor combinations [9]. These represent a variety of drug classes, including carbapenems, β-lactams, cephalosporins, quinolones, tetracyclines, glycylcyclines, aminoglycosides, and dihydrofolate reductase inhibitors.

The raw WGSS data of this strain, generated by the Illumina platform, was downloaded from the NCBI WGS database (NZ_AOCV01000027.1) [10].

Visualisation of results

We exported the results from TreeSeq to tab-separated value files, which were then imported into TIBCO Spotfire [12], a commercially available analysis tool for interactive display of complex data. Data of interest can easily be exported to various output formats for later use.

Supporting Information

S1 Database. ARDB Fasta file. This is the ARDB that we used for this study. For more information about the database see the method section.
(TXT)

S1 Fig. Search results metagenomic stool dataset using BLAST. Supplementary to Fig 1 this is an interactive Krona chart [8]. It details all the found results with BLAST for each accession-number in the ARDB-database within the metagenomic stool dataset (SRS022524.1).
(HTML)

S2 Fig. Search results metagenomic stool dataset using TreeSeq. Supplementary to Fig 1 this is an interactive Krona chart [8]. It details all the found results with TreeSeq for each accession-number in the ARDB-database within the metagenomic stool dataset (SRS022524.1).
(HTML)

S3 Fig. TreeSeq memory footprint. This graph represents the memory use while filling up the tree. On the x-axis is the number of entries derived from the gene sequences in the ARDB as described in the method section. On the y-axis is the memory footprint in bytes. The individual lines represent different read lengths, ranging from 1 (green) to 100 (red) nucleotides. The marked line in the middle represents read length of 60 nucleotides, used in this study.
(TIFF)

S4 Fig. TreeSeq data compression. These graphs represent the data compression that occurs while filling up the tree, which occurs because of (partial) overlap in the nodes of the tree as described in the method section. On the x-axis is the number of entries derived from the gene sequences in the ARDB as described in the method section. On the y-axis is the compression rate. The individual lines represent different read lengths, ranging from 1 (yellow) to 100 (red) nucleotides. Compression rate = 1 - (((number of nodes in tree)/(read length)) * (sum of reads)) The lower graph is a duplicate of the upper graph with a marked line, which represents the read length of 60 nucleotides that we used for this study.
(TIFF)

S5 Fig. TreeSeq results in different settings. Tree-S100 (Speed 100%) is the fast setting in which only the first 60 nucleotides of the raw data are matched to the tree. Tree-S1 (Speed lowest) is the sensitive setting, which also searches within a read until it has a hit. (Left) The sum of occurrences in the different genes in fast and sensitive mode. Note that the sensitive setting leads to more hits. (Right) This scatterplot shows the results distribution for the quaternary tree in the two different settings. Note that the sensitive setting leads to more hits at the low occurring genes but that this method does not lead to a total different distribution.
(EPS)

S1 Software. TreeSeq Terminal version. This is a terminal based version of TreeSeq that will run on Mac OS. This version of the software demonstrates the basic functionality. The ARDB fasta file (S1 Database) in combination with the HMP datasets can be used [7].
(COMMAND)

S1 Table. TreeSeq versus phenotype. American Type Culture Collection (ATCC) tested resistance in Klebsiella pneumonia (BAA-2146), which is a phenotypical referenced strain, by culturing. An overview for detected antimicrobial resistance (1 if tested positive) is shown for culture and TreeSeq.
(XLSX)

Author Contributions

Conceived and designed the experiments: BW CV AB. Performed the experiments: BW BB. Analyzed the data: BW BB. Wrote the paper: BW BB CV AB.

References

1. Didelot X, Bowden R, Wilson DJ, Peto TEA, Crook DW. Transforming clinical microbiology with bacterial genome sequencing. Nat Rev Genet. 2012; 13: 601–612. Available: http://www.nature.com/nrg/journal/v13/n9/full/nrg3226.html. doi: 10.1038/nrg3226 PMID: 22868263
2. Chan JZ, Pallen MJ, Oppenheim B, Constantinidou C. Genome sequencing in clinical microbiology. Nat Biotechnol. 2012; 30: 1068–1071. Available: http://www.nature.com.ezp-prod1.hul.harvard.edu/nbt/journal/v30/n11/full/nbt.2410.html\nhttp://www.nature.com.ezp-prod1.hul.harvard.edu/nbt/journal/v30/n11/pdf/nbt.2410.pdf. doi: 10.1038/nbt.2410 PMID: 23138305
3. Turnbaugh PJ, Ley RE, Hamady M, Fraser-Liggett CM, Knight R, Gordon JI. The human microbiome project. Nature. 2007; 449: 804–810. Available: http://www.pubmedcentral.nih.gov/articlerender.fcgi?artid=3709439&tool = pmcentrez&rendertype = abstract. PMID: 17943116
4. Peterson J, Garges S, Giovanni M, McInnes P, Wang L, Schloss JA. The NIH Human Microbiome Project. Genome Res. 2009; 19: 2317–2323. Available: http://genome.cshlp.org/content/19/12/2317.long. doi: 10.1101/gr.096651.109 PMID: 19819907
5. Altschul SF, Madden TL, Schäffer AA, Zhang J, Zhang Z, Miller W. Gapped BLAST and PSI-BLAST: a new generation of protein database search programs. Nucleic Acids Res. 1997; 25: 3389–3402. Available: http://www.pubmedcentral.nih.gov/articlerender.fcgi?artid=146917&tool = pmcentrez&rendertype = abstract. PMID: 9254694
6. Liu B, Pop M. ARDB–Antibiotic Resistance Genes Database. Nucleic Acids Res. 2009; 37: D443–7. Available: http://www.pubmedcentral.nih.gov/articlerender.fcgi?artid=2686595&tool = pmcentrez&rendertype = abstract. doi: 10.1093/nar/gkn656 PMID: 18832362
7. Illumina Short Read Archive. Illumina website. Available: http://www.hmpdacc.org/HMASM/. Accessed 4 December 2014.
8. Ondov BD, Bergman NH, Phillippy AM. Interactive metagenomic visualization in a Web browser. BMC Bioinformatics. 2001; 12: 385. Available: http://www.biomedcentral.com/1471-2105/12/385.
9. ATCC-antimicrobials-strains. ATCC website. Available: http://www.atcc.org/~/media/PDFs/Multidrug%20Resistant%20%20Antimicrobial%20Strains.ashx. Accessed 4 December 2014.
10. SRR931757. NCBI website. Available: http://www.ncbi.nlm.nih.gov/sra/SRR931757. Accessed 4 December 2014.
11. Bradford PA. Extended-Spectrum β-Lactamases in the 21st Century: Characterization, Epidemiology, and Detection of This Important Resistance Threat. Clin Microbiol Rev. 2001; 14: 933–951. Available: http://www.ncbi.nlm.nih.gov/pmc/articles/PMC89009/. PMID: 11585791
12. TIBCO Spotfire 6.0. TIBCO website. Available: http://spotfire.tibco.com/. Accessed 4 December 2014.
