# Supplementary material for: Correction: TreeSeq, a Fast and Intuitive Tool for Analysis of Whole Genome and Metagenomic Sequence Data
Source: PLoS One. 2015 Jul 20;10(7):e0133816. doi: 10.1371/journal.pone.0133816 (PMC4508097; doi:10.1371/journal.pone.0133816)
Supplement: S2 File — (PDF) [file pone.0133816.s002.pdf]

CORRECTION

Correction: TreeSeq, a Fast and Intuitive Tool for Analysis of Whole Genome and Metagenomic Sequence Data

Bastiaan B. Wintermans, Bernd W. Brandt, Christina M. J. E. Vandenbroucke-Grauls, Andries E. Budding

The authors' names are listed incorrectly. The correct names are: Bastiaan B. Wintermans, Bernd W. Brandt, Christina M. J. E. Vandenbroucke-Grauls, and Andries E. Budding. The correct citation is: Wintermans BB, Brandt BW, Vandenbroucke-Grauls CMJE, Budding AE (2015) TreeSeq, a Fast and Intuitive Tool for Analysis of Whole Genome and Metagenomic Sequence Data. PLoS ONE 10(5): e0123851. doi:10.1371/journal.pone.0123851

Reference

1. Wintermans B, Brandt B, Vandenbroucke-Grauls C, Budding A (2015) TreeSeq, a Fast and Intuitive Tool for Analysis of Whole Genome and Metagenomic Sequence Data. PLoS ONE 10(5): e0123851. doi: 10.1371/journal.pone.0123851 PMID: 25933115

CrossMark
click for updates

OPEN ACCESS

Citation: Wintermans BB, Brandt BW, Vandenbroucke-Grauls CMJE, Budding AE (2015) Correction: TreeSeq, a Fast and Intuitive Tool for Analysis of Whole Genome and Metagenomic Sequence Data. PLoS ONE 10(6): e0131379. doi:10.1371/journal.pone.0131379

Published: June 22, 2015

Copyright: © 2015 Wintermans et al. This is an open access article distributed under the terms of the Creative Commons Attribution License, which permits unrestricted use, distribution, and reproduction in any medium, provided the original author and source are credited.
